# Hypotension after anesthesia induction in patients taking tricyclic antidepressants—A case series

**DOI:** 10.1111/aas.70001

**Published:** 2025-02-16

**Authors:** Mads Lodsgaard, Birgitte Bech Melchiors, Mogens Krøigaard, Lene Heise Garvey

**Affiliations:** ^1^ Danish Anaesthesia Allergy Centre, Allergy Clinic, Department of Dermatology and Allergy Copenhagen University Hospital Gentofte Denmark; ^2^ Department of Clinical Medicine University of Copenhagen Copenhagen Denmark

**Keywords:** noradrenaline, perioperative anaphylaxis, perioperative hypotension, refractory hypotension, tricyclic antidepressants, vasopressors

## Abstract

**Background:**

Hypotension is commonly observed after induction of anesthesia. Risk factors for intraoperative hypotension include higher ASA class, older age, propofol use, combined general/regional anesthesia, emergency surgery, and use of antihypertensives. Patients who are treated with tricyclic antidepressants (TCAs) may develop severe hypotension in connection with surgery and anesthesia, not responding to vasopressors such as phenylephrine and ephedrine, and use of adrenaline or noradrenaline are necessary to restore the blood pressure. Anaphylaxis may be suspected due to the rapid onset and resistance to usual treatments leading to referral for allergy investigation. The aim of this paper was to identify and describe the clinical characteristics of patients referred to the Danish Anesthesia Allergy Center (DAAC) with perioperative hypotension, without elevation in tryptase, and with negative allergy investigations, who were on regular treatment with TCAs. The pharmacological mechanism behind this phenomenon will also be explored.

**Methods:**

Patients were identified from the DAAC database. Patients with hypotension (systolic blood pressure <75 mmHg) as the only symptom and negative allergy investigations and patients on antidepressants were included. The study period was 2011–2019.

**Results:**

Ten patients were identified. Hypotension occurred after anesthesia induction, the median time from induction to the onset of hypotension was 7.5 min. Eight needed adrenaline or noradrenaline to restore blood pressure. No allergen was identified on detailed investigation and serum tryptase was not significantly elevated.

**Conclusion:**

Monosymptomatic perioperative hypotension without a significant increase in serum tryptase can be caused by TCAs and this is an important differential diagnosis to anaphylaxis. In patients on regular treatment with TCA perioperative hypotension responds well to noradrenaline or adrenaline but less well to vasopressors such as phenylephrine and ephedrine used perioperatively.

**Editorial Comment**

The authors describe a group of patients where a profound decrease in blood pressure occurred after anesthesia induction. Anaphylaxis was suspected but the only explanation seemed to be ongoing treatment with tricyclic antidepressants. The mechanism has not been investigated but blood pressure could be restored with potent vasopressors.

## 1 INTRODUCTION

Perioperative immediate hypersensitivity (POH) reactions, including anaphylaxis, are rare events. However, diagnosis and subsequent allergy investigation are challenging, due to a wide range of drugs administered perioperatively and several important non‐allergic differential diagnoses.^1^ The incidence of POH reactions differs between countries but ranges from one in 18,600 to one in 353 anesthesias and shows considerable geographical variability with regard to the different drugs or substances involved in the reactions.^2^ Hypotension after induction of anesthesia is commonly observed and may lead to postoperative morbidity, including increased risk of acute kidney injury, myocardial injury, tubular dysfunction, and ICU admission.^3^ Risk factors for intraoperative hypotension include higher ASA class, older age, propofol use, combined general/regional anesthesia, emergency surgery, and use of antihypertensives.^3^ Several important non‐allergic differential diagnoses mimic symptoms of perioperative hypersensitivity and relative overdose of anaesthetic drugs, vasodilatory effect of neuraxial blockade, uncontrolled bleeding and other types of shock have been reported as potential differential diagnoses to isolated perioperative hypotension without an increase in serum tryptase.^1^, ^4^, ^5^ Some patients present with severe hypotension which is refractory to routine sympathomimetic drugs (e.g., phenylephrine and ephedrine) and noradrenaline or adrenaline is often required to restore the blood pressure to levels acceptable for the individual patient depending on comorbidities and surgical procedure. In these patients skin symptoms or respiratory symptoms are usually absent, and if tryptase is measured it is not elevated as would be expected in perioperative anaphylaxis. Using the modified Ring & Messmer Scale, these hypotensive episodes fulfil the criteria for anaphylaxis,^5^ but subsequent comprehensive allergy investigation fails to establish a culprit agent. In some cases, it may be observed that patients receive treatment with tricyclic antidepressants (TCAs) on various treatment indications. This clinical phenomenon of perioperative hypotension, typically at induction, in patients treated with TCAs has been described in the literature in a few case reports.^6^, ^7^, ^8^, ^9^, ^10^, ^11^, ^12^ TCAs are predominantly used for treating depression or as secondary analgesics usually administered in lower doses than those used to treat depression.^13^ The aim of this study was to identify and describe the clinical characteristics of patients referred to the Danish Anesthesia Allergy Center (DAAC) who had perioperative hypotension, without elevation in tryptase and with negative allergy investigations, who were on regular treatment with TCAs. The pharmacological mechanism behind this phenomenon is not clearly established and will also be explored in this paper.

## 2 METHODS AND MATERIALS

### 2.1 Approvals

This study was conducted as a retrospective study using data from an existing project “Perioperative allergy⸺optimizing diagnostics, investigation and treatment” approval no P‐2022‐317. The study period was from 2011 to 2019. Patient consent for the publication of data was obtained.

### 2.2 Allergy investigation

DAAC is the Danish national reference center for the investigation of suspected perioperative allergic reactions.^14^ Investigations comprise testing with all intravenous drugs administered within 1 h of the reaction and other drug exposures, for example, intramuscular, subcutaneous, spinal, epidural and local administered within 2 h of the onset of reaction.^15^ Chlorhexidine, latex, ethylene oxide, excipients and lidocaine gel are tested in all patients due to the high probability of exposure in the perioperative setting.^16^ Most drugs and substances are tested in several test modalities comprising in‐vitro tests (Histamine Release Test and specific IgE‐measurement), skin prick test (SPT), and intradermal test (IDT). If other tests are negative, a three‐step provocation test with increasing concentrations of the given drug is performed.^17^ Serum tryptase sampled following the suspected anaphylactic reaction is compared to subsequent baseline serum tryptase typically taken at the time the patient comes for allergy investigation. If the perioperative serum tryptase is >[2 + (1.2 × baseline serum tryptase level)] then the reaction serum tryptase measurement is concluded to be significantly elevated supporting the diagnosis of a perioperative allergic reaction.^18^

### 2.3 Identification of patients

**FIGURE 1:**
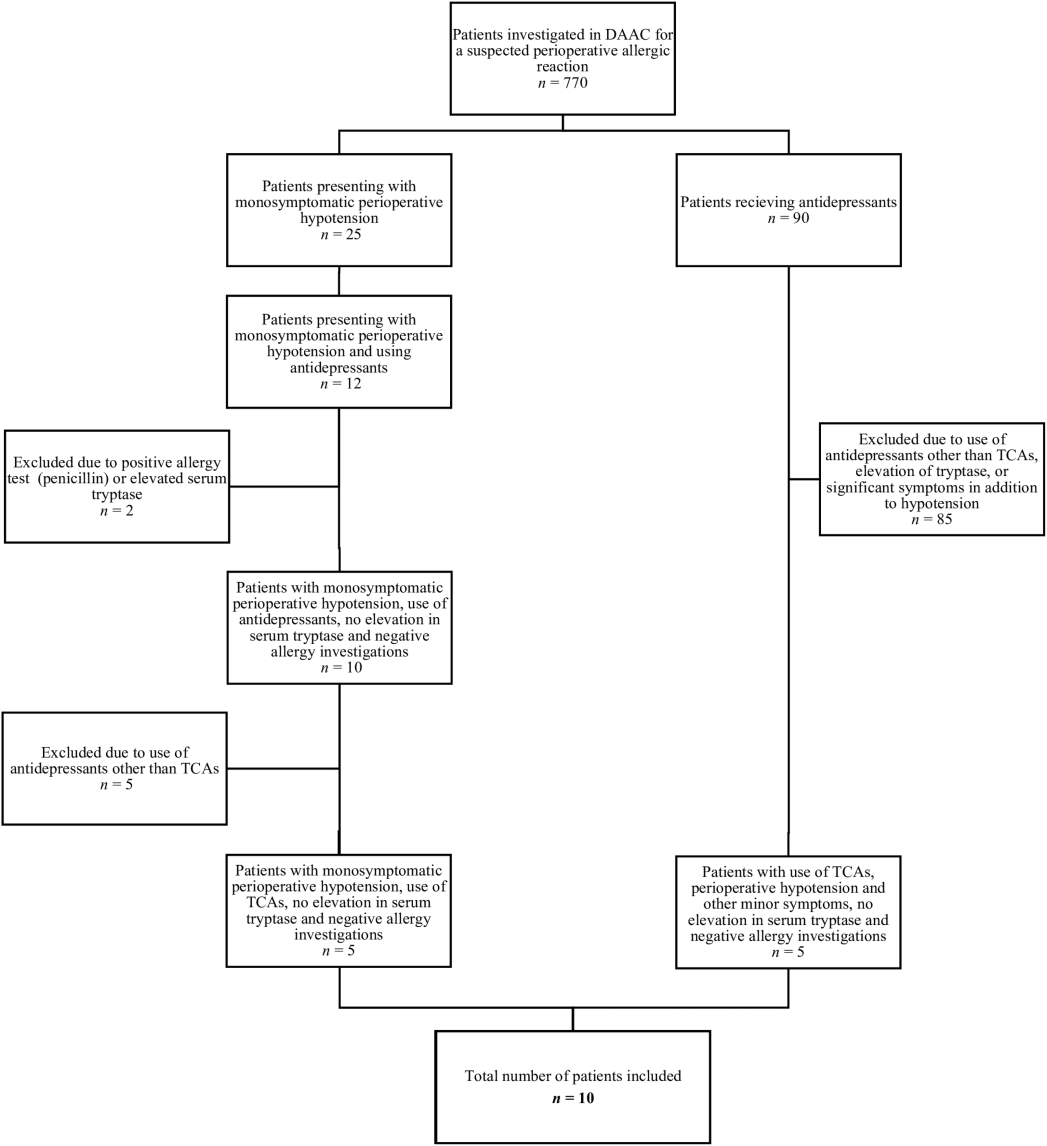
Flowchart of patient inclusion. DAAC, Danish Anesthesia Allergy Center; TCAs, tricyclic antidepressants.

At the time of the study, the DAAC database held data from 770 patients who had undergone investigation for a suspected perioperative allergic reaction. The database contains detailed information regarding prior medical history, the suspected perioperative reaction and results from the allergy investigation. Patients were identified in two ways (Figure 1): First, by searching the database for patients with hypotension as the only symptom and negative allergy investigations. Hypotension is defined as a systolic blood pressure <75 mmHg in the DAAC database. Second, by searching the database for patients on antidepressants. Ten patients fulfilled the inclusion criteria of having perioperative hypotension without elevation in tryptase, negative allergy investigations, and regular treatment with TCAs.

## 3 RESULTS

Of the 10 included patients, six were male and four female (mean age 62 years, range 49–73 years). A summary of clinical characteristics and investigation results can be found in Table 1. Eight patients were classified as ASA II.^19^ All reactions were grade III according to the modified Ring & Messmer Scale, fulfilling the criteria for anaphylaxis.^5^ None of the patients had significantly elevated reaction serum tryptase levels compared to baseline making anaphylaxis less likely.^20^ The hypotensive reactions occurred during general anesthesia for a wide range of surgical procedures and led to the cancellation of the surgery in half of the cases. All reactions happened after induction of anesthesia and the median time from induction to the onset of hypotension was 7.5 min. Five patients took amitriptyline and the remaining five took either nortriptyline, clomipramine, imipramine, or doxepin within the recommended daily dosage.^21^ Four patients had TCAs for depression and six had TCAs as a secondary analgesic. Eight out of ten patients received either adrenaline or noradrenaline to restore their blood pressure. The remaining two were treated with phenylephrine and ephedrine. Five patients (patients 6–10) experienced other minor symptoms in addition to hypotension, which could indicate allergic aetiology. Two patients (patients 6 and 7) developed a diffuse erythematous rash after initial hypotension. Both subsequently underwent surgery; one was anaesthetized using the same drugs and the other patient was anaesthetized without propofol and both patients again developed severe hypotension without skin symptoms. The diffuse rash was therefore regarded as unlikely to have an allergic aetiology and they were both included in the study. Two other patients (patients 8 and 9) experienced localized pruritus around ECG electrodes, and after shaving the genital area in preparation for surgery, respectively. These symptoms were disregarded as systemic allergic symptoms and both patients were included in the study. The last patient (patient 10) developed mild bronchospasm in addition to hypotension. This occurred in the perioperative period after she received neostigmine, which can induce bronchospasm as an adverse effect, and an allergic aetiology was considered unlikely.^21^ Three patients (patients 2, 6, and 7) had experienced more than one similar hypotensive episode during previous or subsequent anesthesias. Anesthesia consisted most frequently of total intravenous anesthesia (TIVA) with propofol and remifentanil. After the onset of hypotension, several patients were changed from propofol and remifentanil to inhaled anesthetics (e.g., sevoflurane or desflurane) and fentanyl or sufentanil.

**TABLE 1:** Clinical Characteristics of patients with severe perioperative hypotension and treatment with a tricyclic antidepressant (TCA).

Patient No.	Sex and age at the time of reaction	TCA treatment indication	Drug and daily dose	ASA class	Surgical procedure	Anesthesia	Perioperative symptoms	Time from induction to onset of hypotension	Ring and messmer scale	Treatment	Tryptase (μg/L)		[2 + (1,2 × baseline tryptase)]	Completion of surgery
											Perioperative	Baseline		
1	M, 57	Depression	Nortriptyline 100 mg	II	Ureteric Stent Placement	PRO, REM, ROC	HT	20 min	III	P, C, IV, NA	^c^	5.54	8.65	Yes
2	M, 62^a^	Neuropathic Pain	Imipramine 10 mg x 2	II	Spine Surgery	PRO, REM, FEN	HT	5 min	III	E, P, IV, A	4.99	5.52	8.62	No
3	F, 57	Tension Headache	Amitriptyline 20 mg	II	Hallux Valgus Surgery	PRO, REM, LID	HT	5 min	III	E, P	9.3	6.28	9.54	No
4	F, 64	Depression	Amitriptyline 75 mg	II	Mastectomy	PRO, REM, FEN, SEV	HT	5 min	III	E, P, A, C, NA	15.4	17.1	22.52	Yes
5	F, 73	Depression	Doxepin 150 mg	II	Cervical Spinal Stenosis Surgery	PRO, REM, ROC	HT	5 min	III	IV, E, P, A	4.76	6.24	9.49	No
6	M, 49^a^	Post‐Infectious Headache	Amitriptyline 75 mg	I	Knee Arthroscopy	PRO, REM, SEV, FEN	HT, R	10 min	III	P, E	3.83	4.23	7.08	Yes
7	M, 67^a^	Restless Legs Syndrome	Clomipramine 10 mg	II	Radical Prostatectomy	PRO, REM, SUX, CIS	HT, R	20 min	III	E, P, HA, A, C	4.24	5.44	8.53	No
8	M, 69^b^	Depression	Nortriptyline 120 mg	II	Radical Prostatectomy	PRO, SUF, ROC, DES	HT, R	15 min	III	E, P, A, HA	3.56	3.46	6.15	No
9	M, 65^b^	Diabetic Neuropathy	Amitriptyline 50 mg	III	Endovascular Aneurysm Repair	PRO, REM, CIS, SEV	HT, PRU	20 min	III	P, E, NA, IV, C, MP	6.39	9.87	13.84	Yes
10	F, 60	Arthralgia	Amitriptyline 25 mg	II	Ureteroscopy	PRO, REM, CIS	HT, B	5 min	III	E, P, A, C, MP	5.16	5.38	8.46	Yes

Abbreviations: Treatment is listed sequentially according to the first drug introduced. A, adrenaline; B, bronchospasm; CIS, cisatracurium; C, clemastine; DES, desflurane; E, ephedrine; FEN, fentanyl; HA, human albumin; HT, hypotension; IV, intravenous fluids; LID, lidocaine; MP, methylprednisolone; NA, noradrenaline; P, phenylephrine; PRO, propofol; PRU, pruritus; R, rash; REM, remifentanil; ROC, rocuronium; SEV, sevoflurane; SUF, sufentanil; SUX, suxamethonium.

^a^ Reactions occurred during more than one anesthesia.

^b^ Serum tryptase obtained >6 h post‐reaction.

^c^ Data not available.

## 4 DISCUSSION

This paper describes profound perioperative hypotension caused by regular treatment with tricyclic antidepressants as an important differential diagnosis to perioperative anaphylaxis. The increasing use of tricyclic antidepressants for depression and frequent off‐label use^22^ underlines the importance of increasing awareness of this differential diagnosis for the anesthetist. Increased awareness of this phenomenon could potentially prevent cancellation of surgery due to misinterpretation of the symptoms as anaphylaxis. A total of 770 patients have been investigated at DAAC and 10 patients (1.3%) were identified who all developed severe hypotension following anaesthetic induction with total intravenous anesthesia. In most cases, hypotension was resistant to treatment with vasopressors such as phenylephrine and ephedrine. They exhibited no or very minor skin symptoms or respiratory symptoms, and serum tryptase taken at the time of reaction was not significantly elevated compared to baseline measurements in the 7 patients where tryptase was available and sampled at the correct time. Adrenaline and/or noradrenaline was required to restore the blood pressure in 80% of the cases. Subsequent allergy investigation failed to identify an allergic cause, and their perioperative hypotension was attributed to treatment with tricyclic antidepressants as a diagnosis of exclusion. This clinical phenomenon has been described in seven case reports.^6^, ^7^, ^8^, ^9^, ^10^, ^11^, ^12^ Unlike the 10 patients investigated in DAAC, none of the patients in the case reports underwent subsequent allergy investigation and serum tryptase measurements were not included. In five of the seven published cases, monosymptomatic perioperative hypotension refractory to vasopressors occurred following anaesthetic induction and treatment with adrenaline and/or noradrenaline was needed to revert the refractory hypotension.^6^, ^7^, ^8^, ^10^, ^12^ As there has been very little focus on TCAs as a cause of perioperative hypotension in the literature, the underlying mechanism is poorly explored. TCAs can be divided into secondary (desipramine, nortriptyline, and protriptyline) and tertiary amines (amitriptyline, clomipramine, doxepin, imipramine, and trimipramine) based on their chemical structure. TCAs exert their actions by inhibiting both the presynaptic reuptake of noradrenaline and serotonin (5‐HT), which leads to the accumulation of the neurotransmitters in the synaptic cleft. Secondary amine TCAs mainly block the reuptake of norepinephrine, whereas tertiary amine TCAs mostly inhibit the reuptake of 5‐HT.^13^ TCAs have a broad range of adverse effects related to off‐target binding to ion channels and receptors. These include binding to muscarinic cholinergic M_1_ receptors, histaminergic H_1_ receptors, sodium channels in the heart, and alpha‐adrenergic *α*_1_ receptors^13^ Continuous treatment with TCAs causes several adaptive changes in physiological function^23^ that have been examined in animal models. Continuous treatment with imipramine results in downregulation of tyrosine hydroxylase. The enzyme tyrosine hydroxylase catalyses the rate‐limiting step in the synthesis of catecholamines and this results in presynaptic depletion of noradrenaline.^24^ Continuous treatment with TCAs leads to the downregulation of postsynaptic *α*‐adrenergic receptors. Consequently, the density of functional *α*‐adrenergic receptors is decreased.^25^ Intravenous anaesthetic drugs such as propofol have a high propensity to induce hypotension following anaesthetic induction due to a decrease in systemic vascular resistance.^26^ The ultrashort‐acting opioid remifentanil is also known to cause hypotension.^27^ The combination of hypotension caused by propofol in combination with remifentanil, and the physiological adaptation caused by TCAs could explain why sympathomimetic drugs such phenylephrine and ephedrine fail to restore the blood pressure in patients who receive TCA treatment. Phenylephrine binds to *α*_1_ receptors and ephedrine works as an *α* and *β* receptor agonist and also enhances the release of endogenous norepinephrine from synaptic neurons.^21^ The physiological alterations caused by TCAs combined with their affinity for α‐adrenergic receptors result in perioperative hypotension following anaesthetic induction. The *α*_1_‐adrenergic receptor antagonism, the decreased density of functional *α*_1_‐adrenergic receptors and the depletion of catecholamine storages explain why phenylephrine and ephedrine fail to restore blood pressure. Treatment with adrenaline or noradrenaline, often used as a last resort, is effective due to the higher receptor affinity for *α*‐adrenergic and *β*‐adrenergic receptors.^21^ Use of inhaled anesthesia at induction, such as sevoflurane, is associated with better maintenance of blood pressure compared to propofol.^28^ Therefore, inhaled anaesthetic gases could be used in these patients for induction and/or for maintenance of anesthesia to prevent hypotension. Fentanyl has minimal effects on haemodynamic function and can therefore also be used.^29^ Discontinuation of treatment with TCAs prior to surgery is not recommended, as it does not affect the incidence of hypotension during anesthesia but carries the risk of worsening symptoms of depression and delirium or confusion.^23^ Instead, when faced with patients on TCAs anaesthetists should be aware of the risk of perioperative hypotension and be prepared to treat it early. The optimal treatment choice in these patients is currently unknown, but the advantageous pharmacodynamic profile of noradrenaline (primarily binding to *α*_1_‐adrenoceptors) compared to adrenaline's nonselective agonism of *α*‐adrenoceptors and *β*‐adrenoceptors^21^ would suggest that noradrenaline should be used as first‐line treatment of perioperative hypotension in patients on TCAs. In addition, noradrenaline could be considered as the first‐line vasopressor in patients taking TCAs undergoing anesthesia. Adrenaline is the first line treatment of perioperative anaphylaxis, and an international consensus paper recommends the following doses of adrenaline: 20 μg IV adrenaline for Grade II, 50–100 μg for Grade III, escalating to 200 μg if needed, and 1 mg for Grade IV (cardiac arrest) as per ALS guidelines.^4^ Another pharmacological approach with a different mechanism of action is terlipressin. Terlipressin works as a V_1_‐receptor agonist, and these receptors are predominately located in smooth muscle cells in blood vessels.^30^ On a case report level, terlipressin has effectively been utilized to treat hypotension refractory to noradrenaline and adrenaline following an acute tricyclic antidepressant intoxication.^31^ In addition, terlipressin has also demonstrated that it is as effective as noradrenaline in improving arterial hypotension after failure of ephedrine in patients under general anesthesia, with a shorter onset and longer duration of action.^28^ In conclusion, regular treatment with tricyclic antidepressants should be considered a potential underlying cause of severe perioperative hypotension in patients exhibiting no other allergy symptoms during the reaction and where tryptase sampled at the correct time is not elevated. The decision about the need for allergy investigation in these patients should be based on careful evaluation of the case and collaboration between an allergist and an anesthetist. In addition, anesthetists should be aware of the potential risk of perioperative hypotension in patients on treatment with TCAs and should be ready to treat hypotension with noradrenaline or adrenaline as first‐line vasopressors.

## AUTHOR CONTRIBUTIONS

ML drafted the first manuscript. LHG, BBM, and MK critically revised the manuscript. LHG, BBM, and MK performed the collection of patient data. All authors approved the final version.

## FUNDING INFORMATION

This research received no specific grant from any funding agency in the public, commercial, or not‐for‐profit sectors.

## CONFLICT OF INTEREST STATEMENT

The authors declare that they have no conflict of interest.

## Data Availability

The data that support the findings of this study are available on request from the corresponding author. The data are not publicly available due to privacy or ethical restrictions.
